# Metabolic studies of *Ogataea polymorpha* using nine different corn steep liquors

**DOI:** 10.1186/s12896-024-00927-5

**Published:** 2025-01-10

**Authors:** Sekar Mayang W. Wahjudi, Dominik Engel, Jochen Büchs

**Affiliations:** Aachener Verfahrenstechnik – Biochemical Engineering, RWTH Aachen University, Forckenbeckstr. 51, 52074 Aachen, Germany

**Keywords:** *Ogataea polymorpha*, RAMOS, µTOM, Online monitoring, Batch-to-batch variation, Corn steep liquor, Media supplementation, Morphology change, Fingerprinting

## Abstract

**Background:**

In the fermentation industry, the demand to replace expensive complex media components is increasing for alternative nutrient sources derived from waste or side streams, such as corn steep liquor (CSL). However, the use of CSL is associated with common problems of side products, such as batch-to-batch variations and compositional inconsistencies. In this study, to detect batch-to-batch variations in CSL for *Ogataea polymorpha* cultivations, a “fingerprinting” system was developed by employing the Respiration Activity Monitoring System designed for shake flasks (RAMOS) and 96-well microtiter plates (µTOM).

**Results:**

At 2.5 g d.s./L CSL and 5 g/L glucose, a limitation by a secondary substrate, other than the carbon source, was observed. For this specific CSL medium, this limitation was caused by ammonium nitrogen and could be removed through targeted supplementation of ammonium sulphate. Under ammonium nitrogen limitation, *O. polymorpha* showed a change in morphology and developed a different cell size distribution. Increasing CSL storage times impaired *O. polymorpha* cultivation results. It was speculated that this observation is caused by micronutrient precipitation as sulfide salts. Through targeted nutrient supplementation, these limiting microelements were identified to be copper, iron and zinc.

**Conclusions:**

This study shows the versatility of CSL as an alternative nutrient source for *O. polymorpha* cultivations. “Fingerprinting” of CSL batches allows for early screening. Fermentation inconsistencies can be eliminated by selecting the better performing CSL batches or by supplementing and improving an inferior CSL prior to large-scale productions.

**Supplementary Information:**

The online version contains supplementary material available at 10.1186/s12896-024-00927-5.

## Introduction

Due to depletion of natural resources, urgent action is required to convert waste and side streams into valuable sustainable resources. Waste valorization has been a continuous trend in biotechnological processes for the past two decades [1]. The use of waste and side streams not only contributes towards a circular economy, but also enables the production of high-quality products at lower cost. In fact, the selection of raw materials for fermentation media still dictates a large part of the total production costs [2]. Biotechnological processes often use costly complex media components, such as yeast extract and peptone, which creates an economic hurdle on industrial scale [3]. This makes agricultural side streams, such as corn steep liquor (CSL), all the more valuable as an economical alternative nutrient source in biotechnological processes [4–7], for substituting costly yeast extract and peptone.

CSL originates from a waste stream in the corn starch wet-milling process. One of the critical steps of corn wet-milling is soaking (“steeping”) corn kernels under controlled aqueous conditions, to prepare them for further milling operations. The steep water is then subjected to an evaporation process to reach a solids content of 40–50% (w/w), with the resulting product termed “corn steep liquor” [8, 9]. CSL is claimed as being rich in organic nitrogen, amino acids, lactic acid, minerals, vitamins and reducing sugars [5, 10]. This richness in nutrients makes CSL a serious contender as an alternative nutrient source in biotechnological processes [11]. The biotechnological use of CSL as an alternative nutrient source covers a wide variety, including the production of penicillin [12], succinic acid [13], enzymes and antibiotics [14]. CSL has been proven beneficial as an additive for many biosynthesized products using different microorganisms, including *Yarrowia lypolitica* for the efficient biosynthesis of erythritol [15, 16] and *Aureobasidium melanogenum* for polymalic acid production [17]. However, the background of CSL originating from a waste stream leads to batch-to-batch variabilities in CSL composition, which is largely influenced by various factors, such as changes in the corn harvest location and steeping process. The resulting inconsistent performance caused by CSL batch-to batch variability presents a major disadvantage for using CSL as a nutrient source for fermentation processes. To ensure a more stable CSL production, there are several options, including the development of a specific production process, where CSL is the main product and not a side stream, as well as post-processing of the CSL coming out from the side stream production. However, as a side stream product, CSL will keep being subjected to batch-to-batch variations. Batch-to-batch variability is a disadvantage that applies not only to side stream products, but also to complex media components, such as yeast extract and peptone. It is reported for yeast extract that different batches can account for up to 50% variation in biomass formation and growth rate [18]. The respiration activity monitoring system (RAMOS) enables a quasi-continuous online measurement of the oxygen transfer rate (OTR), without requiring manual sampling and shaking interruptions. The RAMOS technology monitors changes in gas concentrations and pressure in the headspace of the flasks, and, thereby, calculates gas transfer rates. The RAMOS system has been established for many microbial systems, both anaerobic and anaerobic, as can be found in numerous publications [19–23]. A complete description of its measurement principle can be found in [24, 25]. Diederichs et al. [26] discovered that varying yeast extract batches lead to differences in the production of recombinant enzymes. Similar to yeast extract, it is challenging to analyze the complex matrix of CSL and identify the key components critical to ensure consistent fermentation performance. Elaborate methods for a CSL quality control system have been presented in recent years. Xiao et al. [27] first measured the main components of CSL, including total acidity, total amino acids and total sulfite, by means of NIR spectroscopy and partial least-squares regression. Using UPLC coupled with tandem quadrupole time of flight mass spectroscopy, they later detected six free amino acids present in varying quantities in different batches of CSL [28]. Hofer et al. [29] introduced an HPLC method to quantify nine different B-vitamins in different CSL samples. Still to date, a more convenient method has not been yet presented, which is able to easily and rapidly analyze incoming CSL batches, without the need for extensive screenings.

This study aims to present a fast and reliable method to “fingerprint” CSL batches by means of online monitoring of the oxygen transfer rate (OTR). OTR monitoring is enabled by employing the established RAMOStechnique, designed for shake flask [25, 30] and microtiter plates (µTOM) [23]. Results obtained by the RAMOS technology, designed for shake flasks and MTPs, has been numerously validated by upscaling these results to lab and mid-scale stirred tank fermenters, to mimic industrial conditions [31–34]. In our previous publication, we have shown the applicability of the RAMOS technique between CSL batches in *Escherichia coli* and *Bacillus subtilis* cultivations [35]. A CSL fingerprinting system was established for the respective specific medium composition [35]. In addition, we also investigated the use of CSL as alternative nitrogen source for *Gluconobacter oxydans* (data not shown) and *Corynebacterium glutamicum* (data not shown). In this study, the model microbial system, *Ogataea polymorpha* RB11 pC10-FMD (P_FMD_-GFP), was employed. *O. polymorpha* had been extensively studied with the RAMOS technology and its compatibility with this technique had been proven in numerous publications [23, 25, 36–51]. The RAMOS technique is able to detect varying nutritional properties of different CSL batches by OTR measurement over time. The beneficial effect of supplementations, as well as changes caused by extended CSL storage times are uncovered, which can provide valuable insight when inspecting CSL batches. A fast CSL screening method is established to ensure fermentation consistency.

## Materials and methods

### Media and solutions

All media components were purchased, if not otherwise stated, from Carl Roth GmbH & Co. KG (Karlsruhe, Germany).

In this study, yeast extract-peptone-dextrose (YPD) medium containing 10 g/L yeast extract, 20 g/L peptone and 5 g/L glucose was employed. The pH was adjusted to 6.0 with a 5 M NaOH solution.

Cargill Inc. provided the corn steep liquor (CSL) batches from 2 different locations, as well as market reference batches. All CSL batches show a dry substance content range of 43.8–49.7% (w/w). The raw CSL stock solutions were heat sterilized at 121 °C and 1 bar overpressure for 20 min. Heat sterilized CSL stock solutions were stored at 4 °C. All CSL-based media were immediately prepared prior to experiments, to prevent compositional changes. Stock solutions of glucose, lactate, KH_2_PO_4_, (NH_4_)_2_SO_4_ and MgSO_4_ ⋅ 7H_2_O were sterilized by filtration. A 0.2 μm cutoff filter (VWR International, 0.2 μm PES membrane) was used for all sterile filtrations. 1 M MES buffer solution was prepared by setting its pH to 6.0 with a 5 M NaOH solution, followed by sterile filtration. The CSL-based media were prepared by diluting the sterile CSL stock solution with demineralized water and adding sterile 2-(N-morpholino)ethanesulfonic acid (MES) buffer, glucose and other nutrients, e.g. lactate or (NH_4_)_2_SO_4_, to reach the desired concentrations in the media. The concentrations of CSL and other nutrients were varied depending on the experiment. All CSL-based media contained 0.1 M MES buffer.

For supplementation experiments, single components and/or solutions with mixtures of nutrients based on Syn-6-MES medium were used. The base salts used in the salts supplementation included 1.0 g/L KH_2_PO_4_, 7.66 g/L (NH_4_)_2_SO_4_, 3.3 g/L KCl, 3.0 g/L and MgSO_4_ ⋅ 7H_2_O, 0.3 g/L NaCl. Furthermore, 10 mL/L of a 100 g/L CaCl_2_ x H_2_O solution, 10 mL/L microelements solution, 10 mL/L vitamin solution and 10 mL/L trace elements solution were used as supplements. All stock solutions were sterile filtered. The microelements solution comprises of 6.65 g/L, (NH_4_)_2_Fe(SO_4_)_2_ ⋅ 6 H_2_O, 0.55 g/L CuSO_4_ ⋅ 5 H_2_O, 2.0 g/L ZnSO_4_ ⋅ 7 H_2_O, 2.65 g/L MnSO_4_ ⋅ H_2_O, 6.65 g/L Na_2_EDTA (Titriplex III, Merck, Darmstadt, Germany). The vitamin solution consists of 0.4 g/L D-biotin and 13.35 g/L thiamine chloride hydrochloride, whereas the trace element solution consists of 0.065 mg/L NiSO_4_ ⋅ 6 H_2_O, 0.065 mg/L CoCl_2_ ⋅ 6 H_2_O, 0.065 mg/L H_3_BO_3_, 0.065 mg/L KI and 0.065 mg/L Na_2_MoO_4_ ⋅ 2 H_2_O.

In the following investigations, the crystal water content is deducted from all calculations, so that only the dry salt portion of the micronutrients will be considered in our analysis.

### Microorganisms

This work utilized *Ogataea polymorpha* RB11 pC10-FMD (P_FMD_-GFP) as model organism, producing a green fluorescence protein (GFP) under the control of a formate dehydrogenase (FMD) promoter.

### Cultivation procedure

All cultivation experiments were performed in an incubator shaker (ISF1-X, Kuhner Shaker GmbH Herzogenrath, Germany), at constant cultivation conditions (temperature and humidity).

*Ogataea polymorpha* RB11 pC10-FMD (P_FMD_-GFP) was cultivated in YPD medium for preparing cryo cultures. As shown in our previous publications, growth rates can be calculated by measuring oxygen transfer rate (OTR) over time [52, 53]. *O. polymorpha* was monitored online by the RAMOS technology, with cells being harvested during the late exponential growth phase. Upon harvesting, cells were suspended in a 50% (v/v) glycerol/cell suspension ratio of 3/7 (v/v) in 2 mL vials at − 80 °C.

For pre-cultures, the above-mentioned cryo culture was used to inoculate the freshly prepared CSL medium at a ratio of 1/1000. Pre-cultures were monitored in shake flasks via a RAMOS device. Once an oxygen transfer rate (OTR) of > 20 mmol/L/h was reached in the late exponential growth phase, cells were harvested to inoculate the main culture at an initial optical density (OD) at 600 nm of OD_600_ = 0.1. This inoculation ratio was chosen to ensure an appropriate cell density. The chosen ratio enables a sufficient duration of the exponential increase of the microbial activity and, correspondingly, sufficient numbers of measurements.

Depending on the experiment, the main culture is conducted as shake flask cultivation or microtiter plate cultivation.

All pre-cultures, as well as all shake flask cultivations, were conducted in modified 250 mL shake flasks at a filling volume of 10 mL. Further cultivation conditions include a shaking diameter of 50 mm, a shaking frequency of 350 rpm and constant temperature of 37 °C.

Furthermore, microtiter plate cultivations were performed in 96-round well microtiter plates (850301, HJ-BIOANALYTIK GmbH, Erkelenz, Germany) at 37 °C with a shaking diameter of 50 mm and a shaking frequency of 350 rpm. The initial filling volume of the microtiter plates was maintained at 200 µL for all microtiter plate cultivations. Moreover, to ensure sterile conditions, “AreaSeal Film” sealings (A9224, Sigma-Aldrich Chemie GmbH, Germany) were utilized as a sterile barrier in all microtiter plate cultivations.

### Online measurements of oxygen transfer rate (OTR)

All cultivation experiments were conducted in the in-house developed respiration activity monitoring system (RAMOS) [25], which allows an online monitoring of respiratory activity for all cultivations on 8 positions of the device. The precultures and shake flask cultivations were performed in modified 250 mL shake flasks. The respiration activity in microtiter plates allows for measurements in all 96 wells and is monitored by the newly developed µTOM device. The µTOM enables the measurement of the respiratory activity in each individual well of a 96-well microtiter plate [23].

### Sample analysis

For offline analysis in time-resolved experiments, samples were obtained from conventional shake flasks equipped with cotton plugs, which are cultivated together and under identical conditions as the RAMOS flasks. The conventional shake flasks were removed from the incubator once the cultivation broth had been taken as sample at a specific time point. It has been proven in the past that cultivations in RAMOS and conventional shake flasks show the same kinetic behavior [54]. For all other experiments, only final samples were taken at the end of cultivation.

pH measurements were carried out with InLab Easy pH electrode (Mettler Toledo, Germany), equipped with a Cyber-Scan pH 510 m device (Eutech Instruments, Thermo Scientific, Germany). A photometer (Genesys 20, Thermo Scientific, Germany) was used to measure the optical density at a wavelength of 600 nm. Samples from cultivation experiments were diluted with a 0.9% NaCl solution to achieve a measurement range of 0.1–0.3 in the photometer, maintaining the ideal linear range of OD_600_ measurements. The CSL-based media contain solid particles and, thus, generate an initial optical density. Therefore, the initial optical density of every CSL-based medium was subtracted from the final OD_600_ measurements. Residues of the carbon sources glucose and lactate were measured by high-pressure liquid chromatography (HPLC) (Ultimate, Dionex, Germany) using an organic acid resin column (300 × 7.8 mm, Phenomenex, Inc., Germany) and a refractometer for detection (Shodex RI-01, Shodwa Denko Europe, Germany). As eluent, 5 mM H_2_SO_4_ was employed at a flow rate of 0.8 mL/min and 60 °C. Lactic and acetic acid were measured by high-performance liquid chromatography (HPLC) (Waters Alliance, Waters Chromatography Europe B.V., The Netherlands), employing two organic acid resin columns in series (30 cm x 8 mm, Waters Shodex KC-811 H^+^, Waters Chromatography Europe B.V.), as well as a refractometer for detection (Waters 2414 Refractive Index Detector, Waters Chromatography Europe B.V., The Netherlands). 10 mM H_2_SO_4_ served as an eluent at a flow rate of 0.5 mL/min and 60 °C. Primary amino acids were derivatized with diethyl ethoxymethylenemalonate (DEEMM) and separated by reversed-phase HPLC using a C18 column (Waters Acquity UHPLC Class H, Waters Chromatography Europe B.V., The Netherlands). Detection of the analytes was performed by UV absorbance (Thermo Scientific Ultimate 300 RS Diode Array Detector, Thermo Fisher Scientific Inc., USA) at 282 nm, quantifying the analytes by comparing the peak area of each analyte with the corresponding standard. The total nitrogen content was measured by the Dumas method, performed with a TruMac C/N analyser (LECO Instruments UK Ltd., UK). The total phosphorus content was determined by inductively coupled plasma atomic emission spectroscopy (ICP-AES), using a PerkinElmer Optima 2000 DV device (PerkinElmer Instruments, USA).

A Coulter Counter 4 device (Beckman Coulter, Krefeld, Germany) was used to determine cell concentrations based on particle size distribution measurements. Prior to measurements, samples were prepared by dilution with Isoton II solution (Beckman Coulter) to not exceed the manufacturer’s declared particle concentration threshold of 10% of the total particle concentration, thus, minimizing the occurrence of artifacts. For measurements, 0.1 mL of the pre-diluted sample was mixed with 19.9 mL Isoton II solution. The measurement range was 0.2–18 μm, using 400 size bins as the highest measuring resolution. Each sample was measured in duplicates.

## Results and discussion

### Metabolic activity of *Ogataea polymorpha* on YPD medium and CSL-based media

*Ogataea polymorpha* RB11 pC10-FMD (PFMD-GFP) was cultivated on the commonly employed YPD medium and on selected CSL-based media. Pure CSL was employed as one of the CSL-based media, in order to examine the potential of CSL as a sole nutrient source for the *O. polymorpha* strain. The carbon sources glucose and lactate, respectively, were added to CSL medium to test the applicability of CSL as a nitrogen source for this cultivation system. Since introducing an additional waste material (e.g. molasses or pectic substrates) as a cheaper carbon source substitute would add more variation into the medium composition and shift the focus away from the role of corn steep liquor as a nitrogen source, pure glucose was used as carbon source. Cheaper carbon source substitutes, such as molasses [34, 55, 56], thin and thick juice [34] from the sugar manufacturing process, and pectic substrates such as polygalacturonic acid [57], were investigated in our previous publications. The metabolic activity of the *O. polymorpha* strain on the different media was compared.

**Fig. 1:**
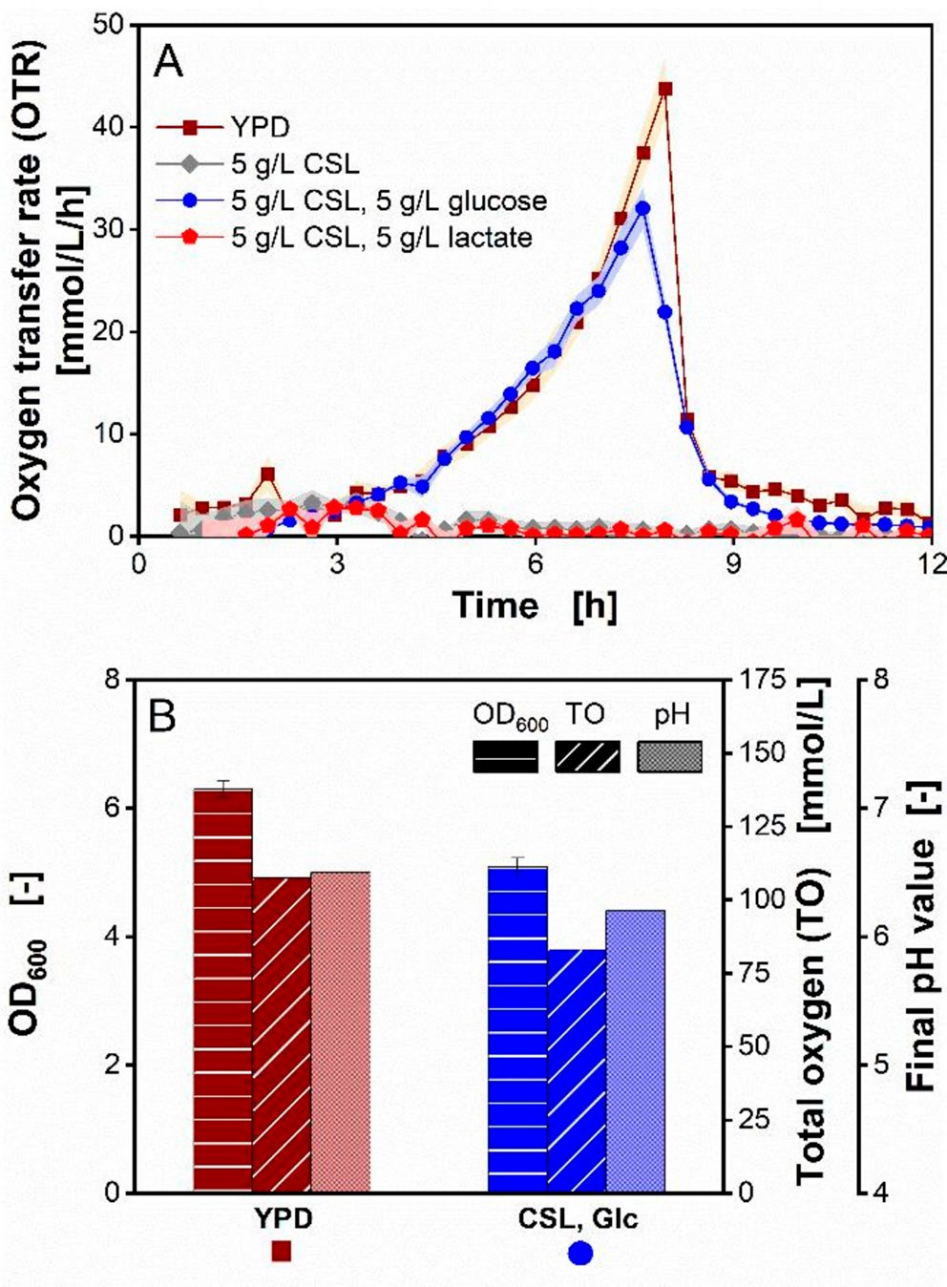
Characterization of *Ogataea polymorpha* metabolic activity on yeast extract-peptone-dextrose (YPD) based media and corn steep liquor (CSL) based media in RAMOS flasks. (**A**) Oxygen transfer rate of *O. polymorpha* RB11 pC10-FMD (P_FMD_-GFP). Data points shown are mean values, ± half the amplitude between duplicate cultivations is shown in shadows. (**B**) Optical density at 600 nm wavelength (OD_600_), total oxygen (TO) and final pH values, measured after 18 h. OD_600_ and final pH values were obtained in triplicates, whereas the mean value for TO was calculated from two OTR values from duplicate cultivation. Error bars for OD_600_ and final pH values in (**B**) indicate the respective standard deviation. Error bars for pH values are barely visible, due to low standard deviations < 0.5. Experimental parameters: 250 mL RAMOS flask; YPD medium = 10 g/L yeast extract, 20 g/L peptone, 5 g/L glucose; CSL medium = 5 g/L CSL 1, 0.1 M MES buffer; glucose concentration $\:{c}_{glucose}$ (if applied) = 5 g/L, $\:{c}_{lactate}$ (if applied) = 5 g/L, initial pH = 6.0, culture volume V_L_ = 10 mL, shaking frequency *n* = 350 rpm, shaking diameter d_0_ = 50 mm, temperature T = 37 °C and initial OD_600_ = 0.1

The metabolic activities of *O. polymorpha* on YPD medium and CSL-based media are displayed in Fig. 1. As shown in Fig. 1A, cultivating *O. polymorpha* in the different media resulted in significant OTR course differences. When grown in YPD medium containing 10 g/L yeast extract, 20 g/L peptone and 5 g/L glucose, a steep increase of OTR is observed, reaching a maximum OTR of 44 mmol/L/h (Fig. 1A). During growth on YPD medium, the total oxygen (TO) consumed accumulated to 108 mmol/L. The final pH measured at the end of the cultivation on YPD medium was 6.5 (Fig. 1B). This is consistent with reports by Losen et al. [58], that growth on complex compounds can lead to the release of ammonium ions, which in this case led to an increase in pH from 6 to 6.5 (Fig. 1B). Furthermore, cultivating *O. polymorpha* on YPD medium resulted in a final optical density (OD_600_) of 6.3 (Fig. 1B).

In contrast to growth on YPD medium, cultivating *O. polymorpha* on pure CSL medium, containing only CSL and MES buffer, did not exhibit any significant metabolic activity (Fig. 1). This lack of metabolic activity is reflected by the almost negligible OTR course (Fig. 1A) and the equally negligible OD_600_ (data not shown). Since lactate is the most abundant carbohydrate in CSL [59–61], this result is consistent with findings by Smutok et al., that *O. polymorpha* is unable to utilize lactate as sole carbon source [62].

Adding glucose to the CSL medium allows for growth of *O. polymorpha*, resulting in a steep increase in OTR, identical to growth on YPD medium (Fig. 1A). The OTR increased to a maximum of 32 mmol/L/h, before a steep decline was observed, indicating depletion of the carbon source glucose (Fig. 1A). Growth on glucose-enriched CSL medium led to a TO of 83.2 mmol/L, whereas the final OD_600_ amounted to 5.1 (Fig. 1B).

As can be hypothesized from the cultivation on YPD medium, carbohydrates in the glucose-enriched CSL medium are degraded and metabolized first, followed by metabolism of proteins and amino acids contained in the CSL. This explains the slight pH increase from 6 to 6.2 at the end of the cultivation (Fig. 1B). As was observed in the pure CSL medium, no growth was detected when *O. polymorpha* was cultivated on CSL-lactate medium (Fig. 1A). This result further confirmed that lactate as sole nutrient source is unable to induce the growth of *O. polymorpha*, as reported by Smutok et al. [62].

Compared to the results obtained with YPD medium, the measurement values (max. OTR, TO and OD_600_) obtained in glucose-enriched CSL medium are up to 27% lower. However, only 1/6 of complex compounds is incorporated in glucose-enriched CSL medium (5 g/L CSL), compared with the amount used in YPD medium (10 g/L yeast extract and 20 g/L peptone). Hence, glucose-enriched CSL medium proves more efficient in biomass production considering the significant difference of complex compounds contained in the two media.

### Influence of different glucose/CSL (g/g) ratios on *O. polymorpha*

In the previous chapter, it was shown that *O. polymorpha* can be successfully grown on CSL-glucose medium. Using different concentrations of CSL and a constant glucose concentration, the influence of different glucose/CSL (g/g) ratios was studied. Solely CSL batch 1 was used, an identical batch as in the previous chapter. Glucose was added to all CSL-glucose media at a concentration of 5 g/L, while CSL concentrations between 1.25 and 10 g d.s./L were used. To allow for high experimental throughput, this study was performed in a round 96 deep-well microtiter plate and measured with a µTOM device.

**Fig. 2:**
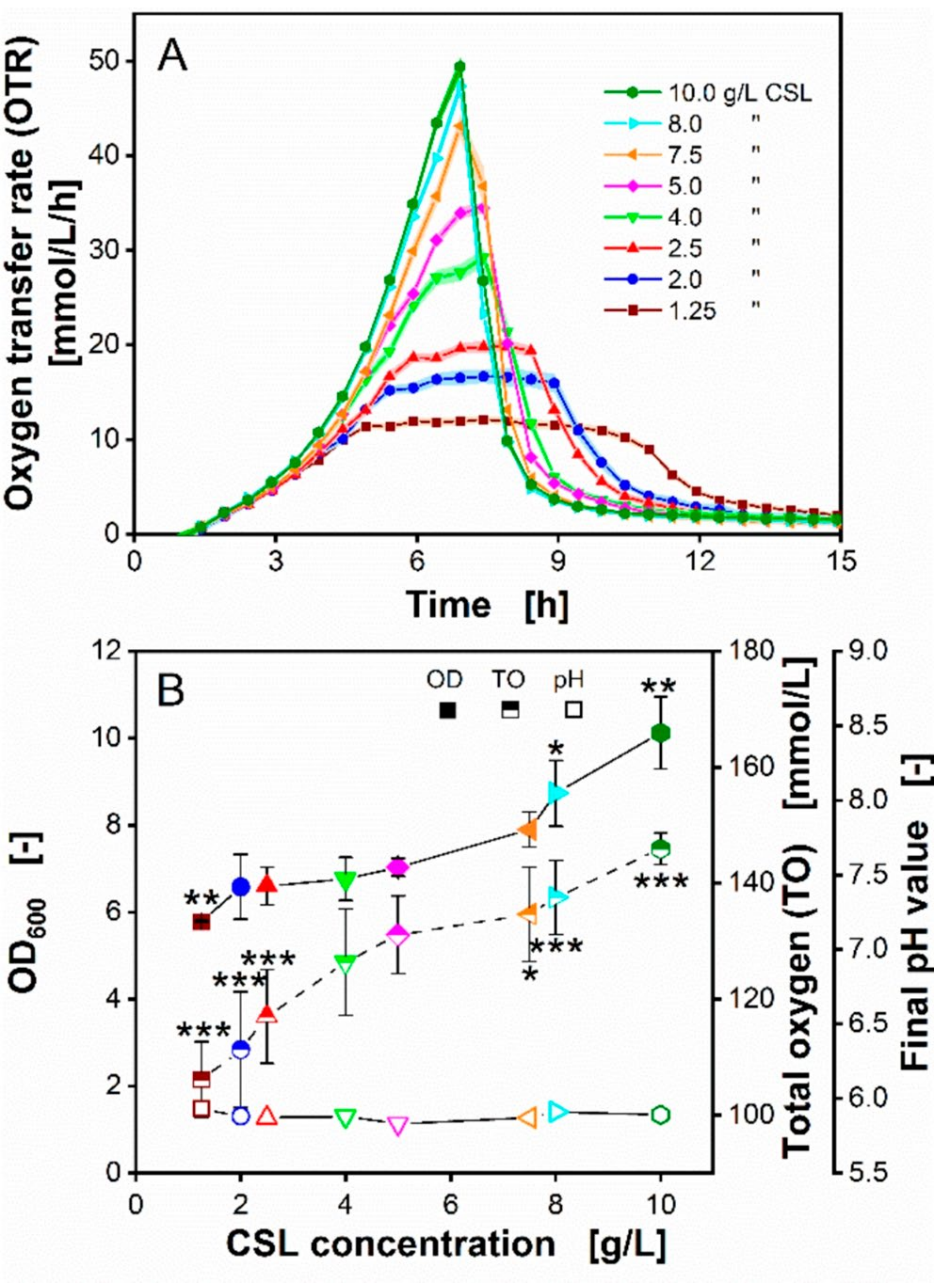
Influence of different corn steep liquor (CSL) 1 concentrations on *Ogataea polymorpha* cultivations in a µTOM device. (**A**) Oxygen transfer rate of *O. polymorpha* RB11 pC10-FMD (PFMD-GFP). All data points are mean values from 11–12 individually measured wells. Shadows indicate the respective standard deviation. For some CSL variants, the respective shadows are barely visible, because of low standard deviations < 0.5. (**B**) The three parameters optical density at 600 nm wavelength (OD_600_), total oxygen (TO) and final pH values, were measured at the end of experiment, after 19 h. OD_600_ and final pH values were obtained in triplicates, whereas the mean value for TO was calculated from OTRs in duplicate cultivations. Error bars for OD_600_ and final pH values in (**B**) indicate the respective standard deviation. Error bars for final pH values are barely visible, due to low standard deviations < 0.5. Significant differences to the reference (5 g/L CSL 1) are marked with asterisks, corresponding to the significance level (* for 0.01 < *p* < 0.05, ** for 0.001 < *p* < 0.01 and *** for *p* < 0.001). Experimental parameters: round deep-well 96-well microtiter plate (MTP), CSL 1 concentration c_CSL,1_ = 1.25–10 g dry substance/L, glucose concentration c_glucose_ = 5 g/L, MES buffer concentration c_MES_ = 0.1 M, initial pH = 6.0, culture volume V_L_ = 200 µL/well, shaking frequency *n* = 350 rpm, shaking diameter d_0_ = 50 mm, humidity = 80%, temperature T = 37 °C and initial OD_600_ = 0.1

Figure 2A presents different OTR patterns, which are attributed to varying CSL concentrations. The different CSL concentrations led to an aligned growth of *O. polymorpha* for the first 3.5 h, after which the OTR diverged. A typical exponential growth, as well as the highest maximum OTR of 49 mmol/L/h were observed using 10 g d.s./L CSL (glucose/CSL ratio of 1/2, g/g), which was followed by a sharp drop of OTR due to glucose depletion (Fig. 2A). A slightly lower maximum OTR was observed at 8 g d.s./L CSL, compared to 10 g d.s./L CSL. However, when the CSL concentration was further reduced, the maximum OTR also decreased significantly. A round peak shape of the OTR pattern is found, when only 5 g d.s./L of CSL was employed. Decreasing the CSL concentration to 2.5 g d.s./L (glucose/CSL ratio of 2/1, g/g), resulted in an OTR plateau with a maximum at around 19 mmol/L/h, which slowly dropped until glucose depletion after 8.4 h (Fig. 2A). This OTR behavior indicates a limitation by secondary substrates, as previously reported [25, 42, 63]. A secondary substrate limitation, such as phosphate or nitrogen, may result in growth stagnation, leading to an extended respiration activity caused by maintenance metabolism [63]. Decreasing the CSL concentration even further to as low as 1.25 g d.s./L (glucose/CSL ratio of 4/1, g/g) resulted in a more severe growth limitation, shown by a lower OTR plateau at 12 mmol/L/h (Fig. 2A).

Figure 2B displays the relation between final optical densities (OD_600_), total oxygen (TO) and final pH value. Significant differences to the reference (5 g/L yeast extract) are marked with asterisks. A p-value < 0.05 was considered significant. Increasing CSL concentrations resulted in higher OD_600_ values. This ties well with our assumption that higher CSL concentrations remove the secondary substrate limitation in *O. polymorpha* (Fig. 2A), thereby improving biomass production. This result is in accordance with findings reported by Huber et al., that a non-limited culture of *O. polymorpha* produced more biomass than its phosphate-limited counterpart [63]. Figure 2B shows that biomass accumulation dynamics are not slowed down when the available carbon is depleted. In a defined minimal medium, microbes will drastically reduce their growth, as soon a secondary substrate limitation is reached. This condition will not occur in a complex medium, because there are readily available substrates and substrates that are available with a time delay. In the case of nitrogen as a limiting component, ammonium is immediately available. Nitrogen from proteins and lipopeptides must first be hydrolytically released. In addition, these processes often have to be induced first. All these effects will, therefore, result in microbes still growing, albeit at a slower rate. Furthermore, increasing CSL concentrations also led to an increase in total oxygen (TO) (Fig. 2B). This TO increase may be explained by the higher concentration of nitrogen associated with high CSL concentrations, as CSL is rich in organic nitrogen [60]. As described by Panikov [64], stoichiometric equations can be used to approximate growth of aerobic heterotrophic microorganisms. Based on the stoichiometric equation for heterotrophic growth, it is evident that more oxygen is required to utilize a higher concentration of nitrogen, which results in a TO increase. As specified in the Material and Methods section, all CSL-glucose media were buffered with 100 mM MES to prevent acidification, commonly associated with growth media containing ammonium as sole nitrogen source [65]. This MES concentration proved sufficient in maintaining the pH at around 6 for CSL concentrations up to 10 g/L, as shown in Fig. 2B.

### Influence of CSL batch-to-batch variations on *O. polymorpha*

To study the CSL batch-to-batch variations, nine different CSL batches were used as the main medium component for *O. polymorpha* cultivations. Out of all CSL concentrations employed in the previous chapter, 2.5 g d.s./L CSL resulted in a clearly recognizable OTR plateau, indicating secondary substrate limitation (Fig. 2A). Therefore, 2.5 g d.s/L of varying CSL batches were enriched with 5 g/L glucose and 100 mM MES buffer. Additional Files 1 & 2 show the difference in lactic acid, acetic acid content and free primary amino acid profiles of the CSL batches, respectively. These differences in composition of the CSL batches might, therefore, result in varying degrees of secondary substrate limitation. This experiment was performed in a round 96 deep-well microtiter plate and measured with a µTOM device.

**Fig. 3:**
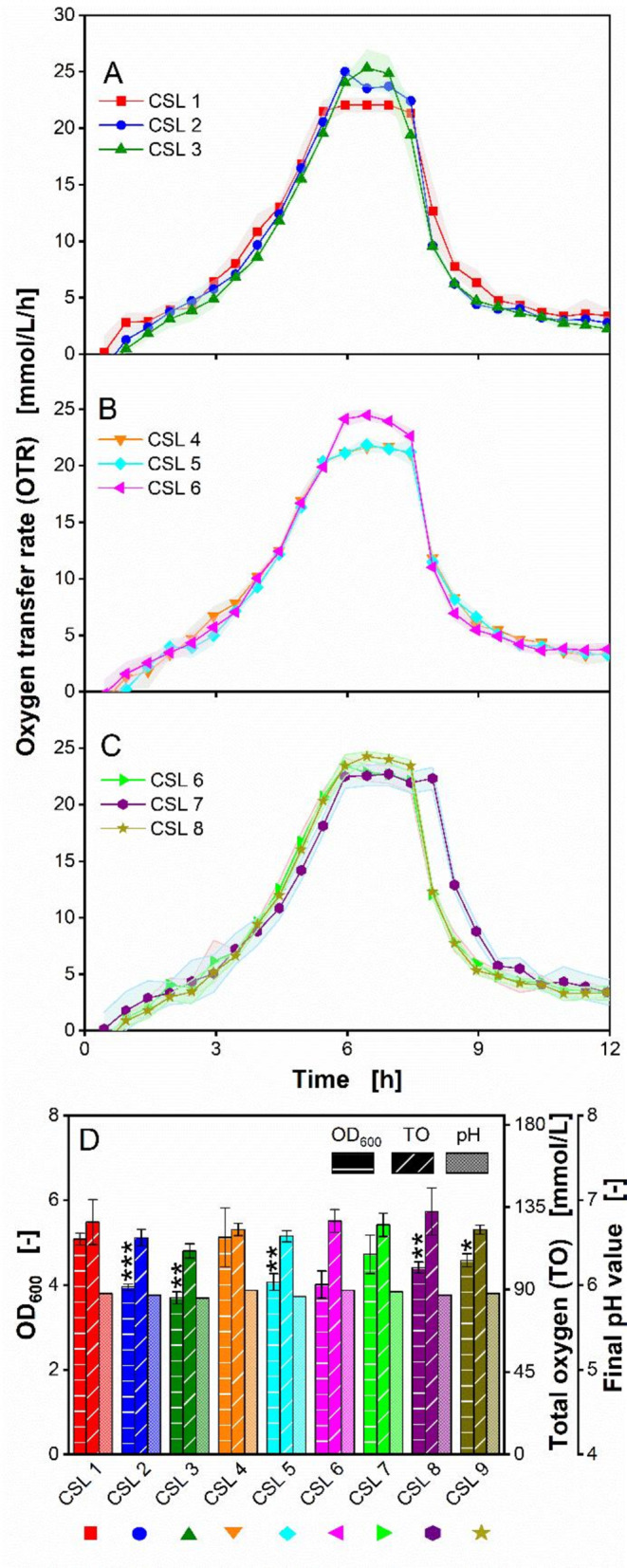
Metabolic activity of *Ogataea polymorpha* during growth on nine CSL variants of different origins (CSL 1–9) in a µTOM device. (**A**-**C**) Oxygen transfer rate of *O. polymorpha* RB11 pC10-FMD (P_FMD_-GFP). Nine different CSL variants are grouped into separate graphs, based on their respective origin (3 production locations). All data points are mean values measured from 4 individual wells. Shadows indicate the respective standard deviation. For some CSL variants, the respective shadows are barely visible, because of low standard deviations < 0.5. The OTR curves are plotted until 12 h, since negligible activity was observed thereafter and all relevant information can be obtained until 12 h. (**D**) The three parameters optical density at 600 nm wavelength (OD_600_), total oxygen (TO) and final pH values, were measured at the end of experiment, after 16.5 h. OD_600_ and final pH values were each obtained from 4 individual wells. TO values were calculated for 4 replicates. Error bars indicate the respective standard deviation. The standard deviations of measured final pH values are low, resulting in barely visible error bars. Significant differences to the reference (2.5 g/L CSL 1) are marked with asterisks, corresponding to the significance level (* for 0.01 < *p* < 0.05, ** for 0.001 < *p* < 0.01 and *** for *p* < 0.001). Experimental parameters: round deep-well 96-well microtiter plate (MTP), CSL 1 concentration $\:{c}_{CSL,1}$ = 2.5 g dry substance/L, glucose concentration $\:{c}_{glucose}$ = 5 g dry substance/L, MES buffer concentration $\:{c}_{MES}$ = 0.1 M, initial pH = 6.0, culture volume V_L_ = 200 µL/well, shaking frequency *n* = 350 rpm, shaking diameter d_0_ = 50 mm, humidity = 80%, temperature T = 37 °C and initial OD_600_ = 0.1

Figure 3 shows the distinct metabolic activities of *O. polymorpha* grown on different CSL batches. The different CSL batches are grouped into three separate graphs, based on their respective local origins (Fig. 3A-C). Similar to the growth of *O. polymorpha* on CSL batch 1 in the previous chapter (Fig. 2A.5 g d.s./L CSL), all nine CSL batches resulted in an aligned OTR increase, exhibiting a plateau, which indicates a secondary substrate limitation (Fig. 3A-C). Between hour 7 and 9, a steep OTR drop was observed, indicating glucose depletion. The differences in OTR behavior of *O. polymorpha* on the varying CSL batches were found to be relatively small. The resulting OTR plateaus ranged between 22 and 25 mmol/L/h, whereas the time point for the OTR drop was identical at 7.5 h for all variants, except for variant 8.

Contrary to expectations, these results prove that *O. polymorpha* is robust to batch-to-batch variations in CSL, despite the use of CSL as the main constituent of the medium, in addition to glucose as a carbohydrate. Our analysis revealed varying concentrations of lactic acid, free amino acid profiles, total nitrogen and total phosphorus in the CSL batches (Add. Files 1–4). The composition of the nine different CSL variants can be found in a table form in Add. File 5. Our previous study on the effect of CSL batch-to-batch variations on *E. coli* showed higher biomass production in cultures that used batches containing a higher concentration of lactic acid [35]. However, since *O. polymorpha* has been shown to be incapable of consuming lactic acid (Fig. 1), the difference in lactic acid content between the CSL batches has little to no effect on its metabolic activity. Furthermore, Klotz et al. showed that a higher concentration of free amino acids and small peptides in yeast extract positively affected the productivity d-lactic acid production in *Sporolactobacillu*s *inulinus* [66]. Different concentrations of free primary amino acids were also identified between CSL batches (Add. File 2), which correspond to reports from Xiao et al. [28]. However, these differences in free primary amino acid concentration also did not significantly impact the metabolic activity of *O. polymorpha*. These results are in contradiction with the results of Zhang et al., showing that different batches of yeast extract markedly affected biomass and antigen productivity [67]. *O. polymorpha* proves robust and less sensitive towards batch-to-batch variations in CSL. Our results may indicate specific characteristics of *O. polymorpha* and can, thus, reflect differences in metabolic pathways between different microorganisms and cultivation conditions. Similar to our observations with *B. subtilis* in our earlier study on CSL [35], it can be inferred that *O. polymorpha* is equally non-susceptible towards changes in CSL variants.

### Ammonium sulfate supplementation removes secondary substrate limitation in *O. polymorpha* cultivations

As previously discussed, cultivating *O. polymorpha* on the employed CSL-glucose medium containing 2.5 g d.s./L CSL and 5 g/L glucose resulted in an OTR plateau, indicating secondary substrate limitation. Therefore, the following objective is to identify the limiting nutrient through targeted supplementation.

Lawford et al. showed that the nutrient requirements of microorganisms can be estimated from the elemental composition of the biomass of these microorganisms [61]. Among other nutrients, it is reported that the macro-nutrients carbon, nitrogen, and phosphorus must be supplied in adequate amounts for microbial growth [61]. For yeasts, Atkinson and Mavituna reported an elemental carbon content of 44.7% (by weight), while the elemental nitrogen content equals 8.5% (by weight) and the phosphorus content totals 1.08% (by weight) [68]. Our results from cultivating *O. polymorpha* on the CSL-glucose medium containing 2.5 g d.s./L CSL and 5 g/L glucose (Fig. 3) yielded a final OD_600_ of 5. With a conversion factor of 0.448 g cell dry weight (CDW)/OD_600_ for *O. polymorpha* from Dusny et al. [69], the CDW of *O. polymorpha* on the employed CSL-glucose medium is calculated to be 2.24 g/L. Based on the previously discussed elemental composition of yeasts from literature [61], our calculated elemental biomass content amounts to 1 g/L for carbon, 0.19 g/L for nitrogen, and 0.024 g/L for phosphorus. Our results from the previous chapters have shown that carbon is abundant in the CSL glucose medium. Therefore, the other two macro-nutrients, nitrogen and phosphorus, are the target of our analysis. Additional File 3 summarizes the total nitrogen content in the CSL variants, which includes ammonium, amino acids, peptides and otherwise bound nitrogen, and ranges from 0.067 to 0.077 (g/g d.s. CSL). Additional File 4 displays the differing phosphorus content in the CSL variants, amounting to 0.035 (g/g d.s. CSL). Since the employed CSL concentration is 2.5 g d.s./L for the investigated CSL-glucose medium, it can be inferred that the nitrogen content in the CSL-glucose medium amounts to 0.17–0.19 g/L, whereas the phosphorus content is estimated to be 0.088 g/L. Taking the calculated biomass composition into account, the nitrogen content in the employed CSL-glucose medium barely meets *O. polymorpha*’s needs (0.17–0.19 g/L versus 0.19 g/L, the latter being the elemental biomass composition of *O. polymorpha*), whereas phosphorus appears to be abundant in the CSL-glucose medium (0.088 g/L versus 0.024 g/L). However, as previously reported by Hofer et al. [70], it is highly uncertain, how much of the compounds contained in CSL are bioavailable. In addition, heat sterilization of CSL as a pre-treatment method may also lead to evaporation of nitrogen and precipitation of phosphorus in CSL, and thus, possible nutrient limitation. Kottmeier et al. investigated the connection between the limitation of magnesium, phosphate and potassium and reduced biomass production in *O. polymorpha* [42]. A limitation of these secondary nutrients resulted in a lowered max. OTR and a reduced biomass production. These observations and estimations were, thus, the motivation for targeted ammonium, magnesium, potassium and phosphorus supplementation.

*O. polymorpha* was cultivated in glucose-enriched CSL medium supplemented with only one type of macro-nutrient at a time. For all CSL media, only CSL batch 1 was utilized. *O. polymorpha* was also grown on a reference CSL-glucose medium without additional supplements. To enable high experimental throughput, these investigations were again conducted in round 96 deep-well microtiter plates and measured by a µTOM device.

Supplementation results of the glucose-enriched CSL medium with 0.1–0.5 g/L magnesium sulfate (MgSO_4_) and 1 g/L potassium dihydrogen phosphate (KH_2_PO_4_) are depicted in Add. File 6 and Add. File 7, respectively. If these nutrients had a positive effect on the OTR and biomass production, it could be concluded that these nutrients were limited in the CSL medium used. However, these negative results prove that none of the nutrients studied (magnesium, sulfur, potassium and phosphorous) is limiting in the CSL-glucose medium used.

**Fig. 4:**
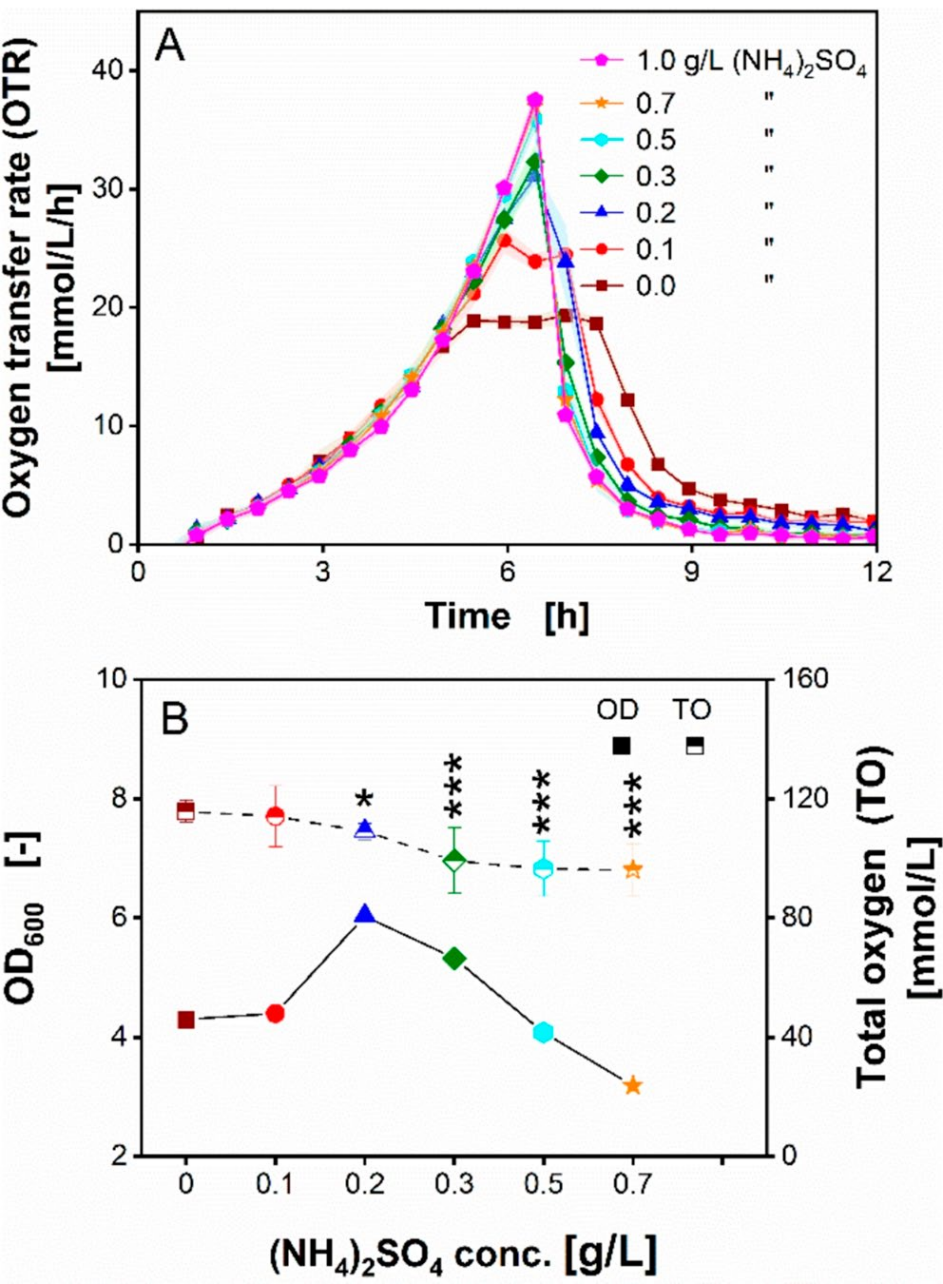
Growth of *Ogataea polymorpha* on CSL 1 medium supplemented with ammonium sulfate in a µTOM device. (**A**) Oxygen transfer rate of *O. polymorpha* RB11 pC10-FMD (P_FMD_-GFP). OTR curves of different ammonium sulfate supplementations. Data points shown are mean values measured from 4–6 individual wells. Shadows indicating the respective standard deviation are barely visible, caused by the low standard deviations < 0.5. (**B**) Optical density at 600 nm wavelength (OD_600_) and total oxygen (TO), measured after 20 h. OD_600_ was each obtained from 2 individual wells. TO values were calculated from OTRs in 5–6 replicates. Error bars in (**B**) for TO values indicate the respective standard deviation. Significant differences to the reference (0 g/L (NH_4_)_2_SO_4_) are marked with asterisks, corresponding to the significance level (* for 0.01 < *p* < 0.05, ** for 0.001 < *p* < 0.01 and *** for *p* < 0.001). Experimental parameters: round deep-well 96-well microtiter plate (MTP), CSL 1 concentration $\:{c}_{CSL,1}$ = 2.5 g dry substance/L, glucose concentration $\:{c}_{glucose}\:$= 5 g/L, ammonium sulfate concentration $\:{c}_{(N{H}_{4}{)}_{2}S{O}_{4}}$ = 0–1 g/L, MES buffer concentration $\:{c}_{MES}$ = 0.1 M, initial pH = 6.0, culture volume V_L_ = 200 µL/well, shaking frequency *n* = 350 rpm, shaking diameter d_0_ = 50 mm, humidity = 80%, temperature T = 37 °C and initial OD_600_ = 0.1

The results of ammonium sulfate ((NH_4_)_2_SO_4_) supplementation to the CSL-glucose medium are depicted in Fig. 4. As shown in Fig. 4A, the addition of only 0.1 g/L of (NH_4_)_2_SO_4_ led to a significantly higher OTR, compared to the reference. Although a plateau-like OTR is still evident at 0.1 g/L (NH_4_)_2_SO_4_, the plateau is completely removed by adding higher (NH_4_)_2_SO_4_ concentrations. Furthermore, the addition of (NH_4_)_2_SO_4_ of more than 0.5 g/L did not lead to further OTR improvement. The highest OTR was reached at 38 mmol/L by supplementing 0.5 and 0.7 g/L (NH_4_)_2_SO_4_, respectively.

Figure 4B presents the dependency of the optical density (OD_600_) and measured total oxygen (TO) on (NH_4_)_2_SO_4_ supplementations. As previously discussed, the reference cultivation led to a substrate limitation-induced OTR plateau (Fig. 4A). This reference cultivation resulted in a final TO of 116 mmol/L (Fig. 4B). As the OTR is improved by the supplementation of higher (NH_4_)_2_SO_4_ concentrations, a decrease of the TO is also observed. Supplementing (NH_4_)_2_SO_4_ concentrations higher than 0.3 g/L decreases the TO to around 96–100 mmol/L. Furthermore, because (NH_4_)_2_SO_4_ concentrations as low as 0.1 g/L improved OTR (Fig. 4A), the final OD_600_ is also expected to increase with higher (NH_4_)_2_SO_4_ concentrations. This correlation is observed at (NH_4_)_2_SO_4_ concentrations of up to 0.2 g/L. However, contrary to expectations, the final OD_600_ decreases, when higher (NH_4_)_2_SO_4_ concentrations were supplemented to the CSL-glucose medium. Judging by the obtained OTR, the supplementation of up to 0.7 g/L (NH_4_)_2_SO_4_ could improve *O. polymorpha* growth, leading to the assumption that these (NH_4_)_2_SO_4_ concentrations are not toxic for the microorganism. There are similarities in the discrepancy of expected growth of *O. polymorpha* between the present study and those described by Kottmeier et al. [51]. In their study, Kottmeier et al. observed that the same *O. polymorpha* strain exhibited higher light scattering intensity under phosphate-limited conditions than its non-limited counterpart. Although they did not encounter significant changes of *O. polymorpha* upon microscopic inspection, flow cytometry measurements revealed two subpopulations as a result of phosphate limitation [51]. Our findings suggest that the same phenomenon of morphological change also occurs under nitrogen limitation. The experiment was repeated using CSL batch 4, which was sourced from a different production location, compared to CSL batch 1. When using CSL batch 4, the decreasing trend of final OD_600_ with increasing (NH_4_)_2_SO_4_ concentrations was much less clear (Add. File 7). At 0.7 g/L (NH_4_)_2_SO_4_, the final OD_600_ was found highest (Add. File 7).

The OTR data demonstrate that non-limited growth of *O. polymorpha* was enabled by supplementing the CSL-glucose medium with (NH_4_)_2_SO_4_ (Fig. 4A). Since magnesium and sulfur could be excluded, because the addition of MgSO_4_ did not improve growth, ammonium nitrogen was identified as the limiting nutrient. Based on the resulting OTR data, a minimum amount of 0.5 g/L (NH_4_)_2_SO_4_ is required to eliminate secondary substrate limitation in this specific CSL-glucose medium. To prevent ammonium nitrogen shortage, the CSL concentration can also be increased in a constant ratio to the applied glucose concentration.

### Ammonium nitrogen limitation affects cell formation of *O. polymorpha*

To further investigate the extent of nitrogen limitation, *O. polymorpha* was cultivated in RAMOS shake flasks on the CSL-glucose medium containing 2.5 g d.s/L CSL and 5 g/L glucose, where it had been shown to be nitrogen-limited (Fig. 4). At the same time with the reference, *O. polymorpha* was also cultivated on a CSL-glucose medium supplemented with 0.6 g/L ammonium sulfate ((NH_4_)_2_SO_4_). Offline samples were taken during cultivation and cell size distribution was measured by a Coulter Counter device.

**Fig. 5:**
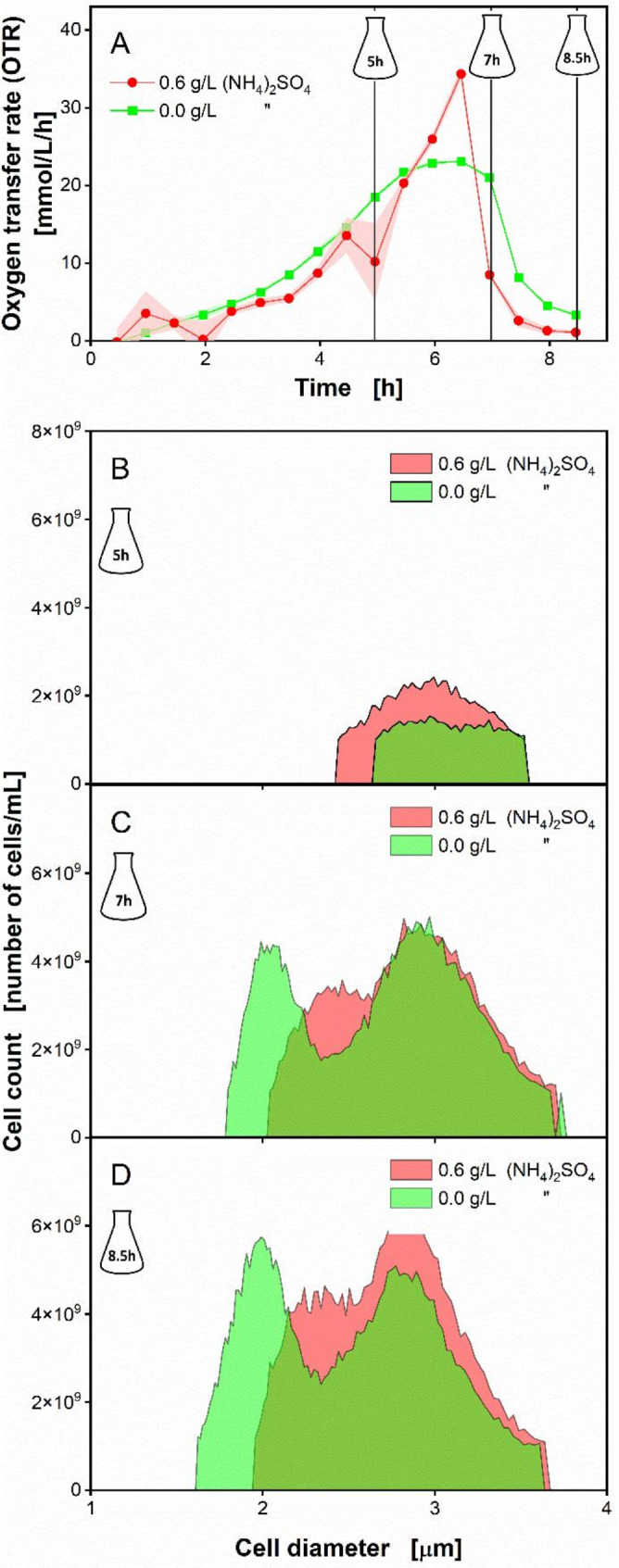
Influence of ammonium limitation on the cell size distribution of *Ogataea polymorpha* cultivations in RAMOS flasks. (**A**) Oxygen transfer rate (OTR) of *O. polymorpha* RB11 pC10-FMD (P_FMD_-GFP). Data points shown are mean values, ± half the amplitude between duplicate cultivations is shown as shadows. OTR was measured until the end of experiment, 8.5 h. (**B**-**D**) Cell size distribution obtained after sampling at hour 5, 7 and at the end of the cultivation (8.5 h). Experimental parameters: 250 mL RAMOS flask, CSL 1 concentration $\:{c}_{CSL,1}$ = 2.5 g dry substance/L, glucose concentration $\:{c}_{glucose}$ = 5 g/L, ammonium sulfate concentration $\:{c}_{(N{H}_{4}{)}_{2}S{O}_{4}}$ = 0 and 0.6 g/L, MES buffer concentration $\:{c}_{MES}$ = 0.1 M, initial pH = 6.0, culture volume V_L_ = 10 mL, shaking frequency *n* = 350 rpm, shaking diameter d_0_ = 50 mm, temperature T = 37 °C and initial OD_600_ = 0.1

Figure 5A shows the OTR of *O. polymorpha* grown on the non-supplemented (0 g/L (NH_4_)_2_SO_4_) and CSL-glucose medium supplemented with 0.6 g/L (NH_4_)_2_SO_4_. The cultivation on the non-supplemented medium led to the expected OTR plateau, which clearly indicates nitrogen limitation, whereas the supplemented culture resulted in a typical OTR pattern without nutritional limitation (Fig. 5A). The higher OTR values in the early stages of the culture without nitrogen supplementation (0 g/L (NH_4_)_2_SO_4_) may be due to slightly different amounts of biomass transferred from the pre-culture to the two different main cultures during inoculation. Offline samples were taken after 5 h, 7 h, and at the end of cultivation (8.5 h). Figure 5B - D display the cell size distribution obtained at the respective sampling time. At the beginning of the experiment (0 h), both cultures showed identical cell size distribution as both were inoculated to an OD_600_ of 0.1 (Add. File 9). At 5 h, nearly overlapping OTR patterns were observed with a slightly higher OTR value measured for the limited culture (0 g/L (NH_4_)_2_SO_4_, Fig. 5A). Figure 5B shows a uniform cell size distribution ranging from 2.6 to 3.6 μm for the limited culture (0 g/L (NH_4_)_2_SO_4_) at t_5_. At the same time, a slightly higher bell-shaped cell size distribution was observed for the non-limited culture (0.6 g/L (NH_4_)_2_SO_4_) ranging between 2.4 and 3.6 μm. At 7 h, the discrepancy in cell size distribution between the two cultures was more prominent (Fig. 5A). Interestingly, both cultures developed a highly similar bell-shaped cell size distribution of around 2.4 and 3.6 μm at 7 h, as well as identical cell count (Fig. 5C). However, the culture supplemented with 0.6 g/L (NH_4_)_2_SO_4_) showed a “shoulder” with a smaller cell size distribution down to 2.02 μm. In contrast, the limited culture (0 g/L (NH_4_)_2_SO_4_) gave a second distinct bell-shaped cell size distribution of smaller cells, at around 1.8 and 2.3 μm, at an equally high cell count of 4.4∙10^9^ (Fig. 5C). At the end of cultivation, 8.5 h, the supplemented culture (0.6 g/L (NH_4_)_2_SO_4_)) revealed a higher concentration of the larger cells (2.4–3.6 μm), while the cell count for the smaller cell fraction at 2.02 μm increased only slightly (Fig. 5D). In comparison, there was no significant increase in cell count of the larger cells (2.4–3.6 μm) in the limited culture (0 g/L (NH_4_)_2_SO_4_). Moreover, the limited culture developed a higher cell count of the smaller cells (1.8–2.3 μm) at 8.5 h (Fig. 5D). In fact, these microcells were also observed under the microscope at the end of cultivation (Add. File 10).

The cell size distribution derived from Coulter Counter measurements (Fig. 5B – D) is in accordance with findings from Boswell et al. [71], who used flow cytometry to detect a bimodal distribution in *Saccharomyces cerevisiae* cells subjected to stress. When *S. cerevisiae* is affected by increased fluid-mechanical stress, Boswell et al. [71] reasoned that smaller single yeast cells and budding yeast cells are formed. Under similar stress conditions, such as the phosphate limitation observed by Kottmeier et al. [51], or the nitrogen limitation in the present study, the same phenomenon of bimodal cell distribution can be observed in *O. polymorpha*. In fact, our analysis revealed a higher number of small cells in the limited culture (Fig. 5D), which corresponds to the results of Porro et al. [72], who reported a correlation between the nutrient content in the applied media and the heterogeneity of yeast cell populations. The morphological change that occurred in *O. polymorpha* is also in agreement with findings from Saldanha et al. [73], who reported a decrease of cell volume in *S. cerevisiae*, when exposed to sulfate limitation.

To obtain a larger sample volume for further investigation, the experiment was repeated in RAMOS flasks. This investigation revealed that the non-supplemented, and, thus, nitrogen-limited culture resulted in a higher OD_600_ (Add. File 11). However, the measured cell dry weight of the nitrogen-limited culture was lower than that of the non-limited culture (Add. File 11). This result supports our prior hypothesis that the measured OD_600_ is misleading in this case, and that the nitrogen-limited culture indeed did not build up more biomass, compared to the supplemented culture, because of apparent growth limitation, as shown by the OTR pattern (Fig. 5A, Add. File 11A). The formation of small cells observed under nitrogen limitation led to an increased OD_600_. Thus, for further studies of yeast cells subjected to nutrient limitation, OD_600_ measurement should not be used as the sole indicator for biomass formation, as the resulting change in cell morphology would most likely render the method inaccurate.

**Fig. 6:**
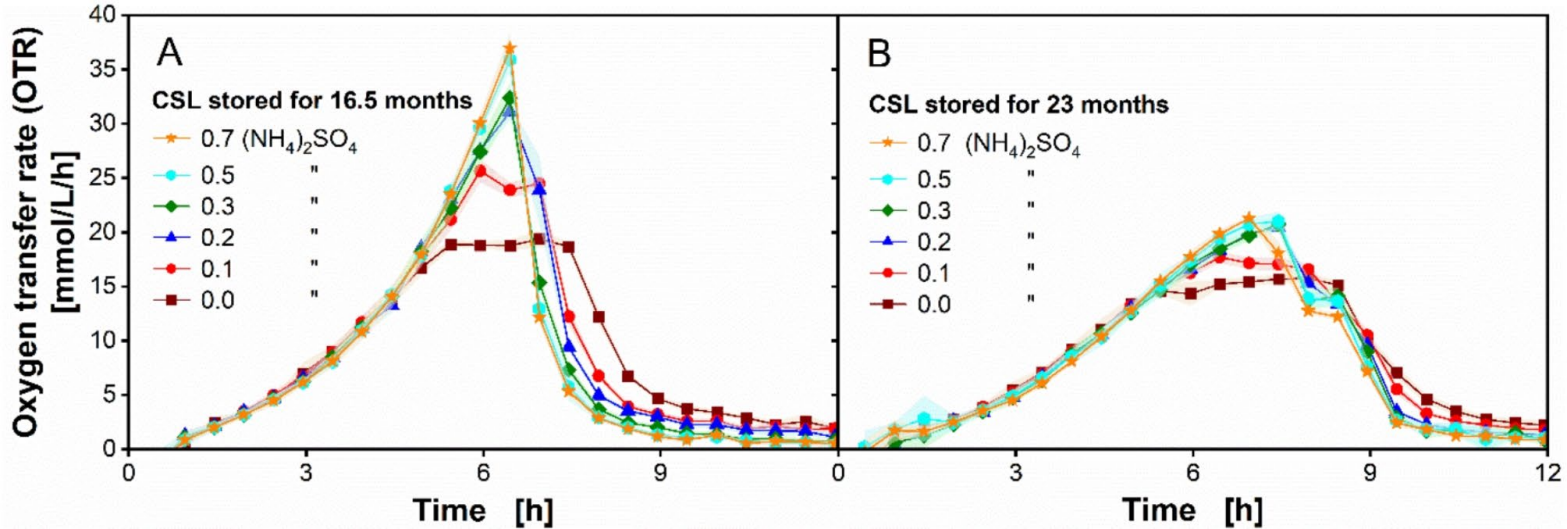
Decreased effect of ammonium sulfate supplementation with longer corn steep liquor (CSL) storage times on *Ogataea polymorpha* in a µTOM device. (**A**-**B**) Oxygen transfer rate (OTR) of *O. polymorpha* RB11 pC10-FMD (P_FMD_-GFP) on CSL medium supplemented with different concentrations of ammonium sulfate. The CSL batch 1 used in (**A**) was stored at 4 °C for 16.5 months. The OTR data in (**A**) are identical to those displayed in Fig. 4A. The data shown in (**B**) were generated using the same CSL batch 1 employed in (**A**), only at a later time, resulting in a storage time of the CSL batch 1 of 23 months. Data points shown are mean values measured from 4–6 individual wells. Shadows indicating the respective standard deviation are barely visible, caused by the low standard deviations < 0.5. Experimental parameters: round deep-well 96-well microtiter plate (MTP), CSL 1 concentration $\:{c}_{CSL,1}$ = 2.5 g dry substance/L, glucose concentration $\:{c}_{glucose}$ = 5 g/L, ammonium sulfate concentration $\:{c}_{(N{H}_{4}{)}_{2}S{O}_{4}}$ = 0–0.7 g/L, MES buffer concentration $\:{c}_{MES}$ = 0.1 M, initial pH = 6.0, culture volume V_L_ = 200 µL/well, shaking frequency *n* = 350 rpm, shaking diameter d_0_ = 50 mm, humidity = 80%, temperature T = 37 °C and initial OD_600_ = 0.1

### Longer CSL storage times decreases the effect of ammonium sulfate supplementation in *O. polymorpha* cultivation

By supplementing ammonium sulfate ((NH_4_)_2_SO_4_), ammonium nitrogen was identified to be the limiting nutrient in the employed CSL-glucose medium. Figure 6A displays the identical OTR results for (NH_4_)_2_SO_4_ supplementation as shown in Fig. 4A. As previously mentioned, the CSL-glucose media were freshly prepared just before inoculation. The OTR data displayed in Fig. 6A were generated by employing a CSL batch 1 stock, stored at 4 °C for 16.5 months. In contrast, Fig. 6B shows the OTR results of an equivalent supplementation experiment, with the exception that the identical CSL batch 1 stock underwent a longer storage time of 23 months. By using CSL batch 1 stored for 16.5 months, the non-supplemented reference cultivation yielded an OTR, which plateaued at 19 mmol/L/h (Fig. 6A). However, with a longer CSL storage time of 23 months, the OTR height of the reference cultivation was reduced by 18% (Fig. 6B). This dramatic effect was evident when comparing different reference cultures containing CSLs stored at different times (Add. File 12). Moreover, a low concentration of (NH_4_)_2_SO_4_, at 0.1 g/L, could result in an immediate OTR improvement, when the CSL storage time was 16.5 months (Fig. 6A). However, the effect of (NH_4_)_2_SO_4_ supplementation dramatically deteriorated with a longer CSL storage time. The highest (NH_4_)_2_SO_4_ concentration of 0.7 g/L was previously sufficient to completely remove the limitation in the CSL-glucose medium (Fig. 6A). By contrast, the same (NH_4_)_2_SO_4_ concentration resulted in little OTR improvement at 21 mmol/L/h in the medium prepared with a longer CSL storage time (Fig. 6B).

These results indicate that ammonium nitrogen is no longer the limiting nutrient for the CSL medium, prepared with a longer stored CSL. Despite storage at 4 °C, it is apparent that a few CSL components became unavailable over time, leading to a poorer performance with longer storage times. Chemical manufacturers often advise that culture media and their components should be stored at a specific temperature and protected from light and dehydration, to avoid compositional change. In addition to microbial infection, components of culture media are also susceptible to chemical degradation [74]. E.g., struvite formation is often present in aqueous culture media and removes available ammonium, magnesium, and phosphate from culture media [75]. Apart from macronutrients, other media components, such as trace metals and microelements, might precipitate and cause performance inconsistencies [76, 77]. Sometimes a coloration of media and media components serves as a signal for chemical alteration, however, as CSL is already colored, that makes it difficult to detect changes. Moreover, since CSL already contains high percentage of solids [78], it is challenging to discern a higher degree of precipitation. Although this study only examined a deteriorating cultivation performance of CSL batch 1, all other CSL batches are expected to experience the same nutrient loss over time, as all CSL batches were pre-treated by heat sterilization and stored under identical conditions.

### Reconstitute CSL performance after long term storage in *O. polymorpha* cultivation by targeted supplementation

**Fig. 7:**
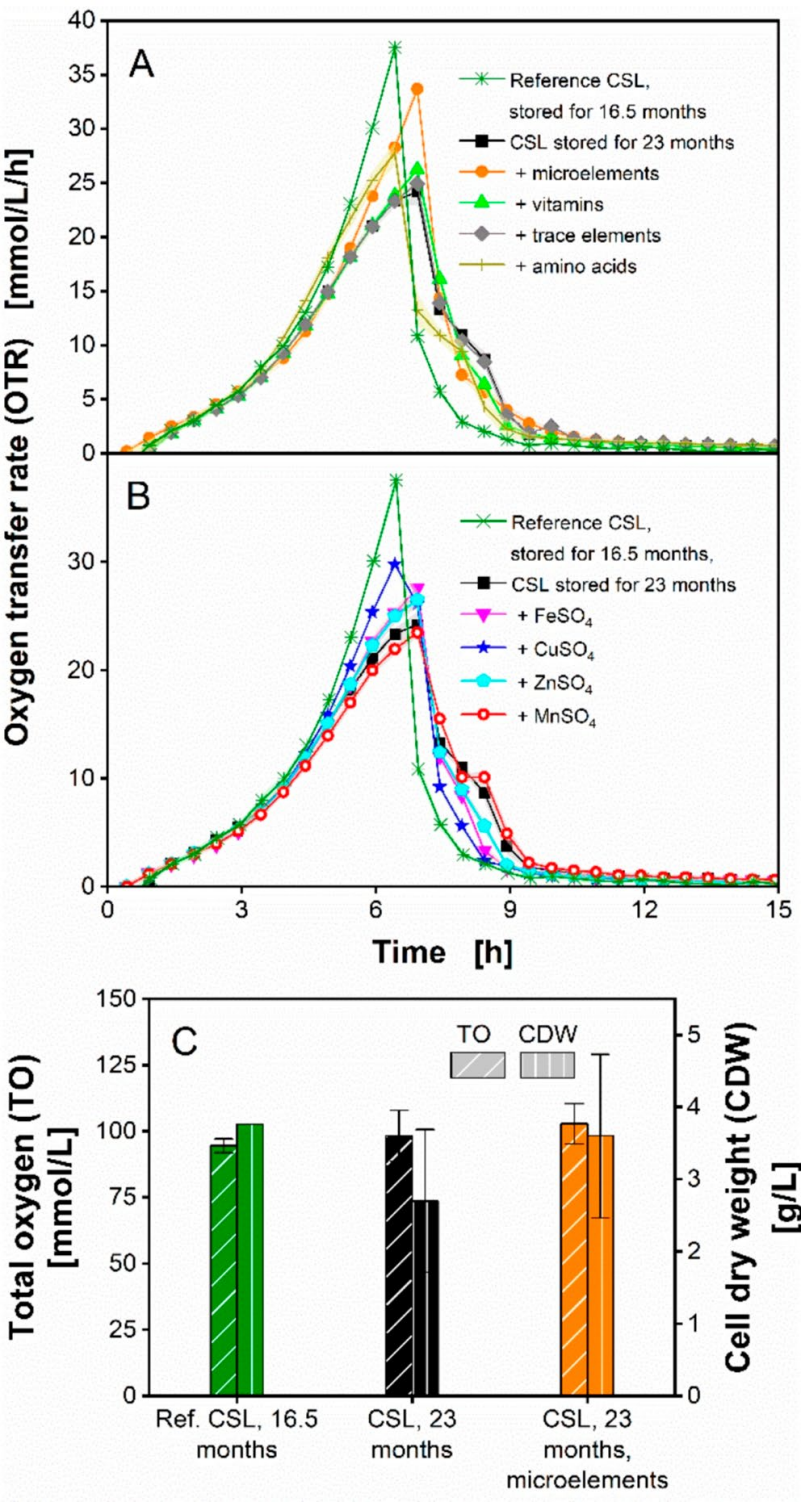
Influence of targeted supplements on the decreased CSL performance after 23 months storage time, measured in a µTOM device. (**A**-**B**) Oxygen transfer rate of *O. polymorpha* RB11 pC10-FMD (P_FMD_-GFP) on CSL medium enriched with different supplements. For clarity, the different supplements are grouped into two separate graphs (**A** and **B**). Shown in A and B are two reference CSL media containing CSL that was stored at different times: 16.5 months and 23 months, respectively. Except for the reference CSL medium containing CSL that was stored for 16.5 months, all other media contained 1.0 g/L ammonium sulfate. (**A**) Effect of different chemical solutions, based on Syn-6 synthetic MES medium, on CSL performance. (**B**) Influence of different compounds of the employed microelements solution on CSL performance. For clarity, the data for MnSO_4_ and ZnSO_4_ are not shown, as the course of the OTRs over time was nearly identical to the reference and the curve for FeSO_4_, respectively. The storage time of the CSL batch employed in (**A**-**B**) was 23 months and the CSL batch was stored at 4 °C. Data points shown are mean values measured from 4–6 individual wells. Shadows, indicating the respective standard deviation, are barely visible, caused by the low standard deviations < 0.5. Experimental parameters: round deep-well 96-well microtiter plate (MTP), CSL 1 concentration $\:{c}_{CSL,1}\:$= 2.5 g dry substance/L, glucose concentration $\:{c}_{glucose}$ = 5 g/L, ammonium sulfate concentration $\:{c}_{(N{H}_{4}{)}_{2}S{O}_{4}}$ = 1.0 g/L, MES buffer concentration $\:{c}_{MES}$ = 0.1 M, initial pH = 6.0, culture volume V_L_ = 200 µL/well, shaking frequency *n* = 350 rpm, shaking diameter d_0_ = 50 mm, humidity = 80%, temperature T = 37 °C and initial OD_600_ = 0.1

To reconstitute the performance of CSL stored for longer periods, a targeted supplementation was implemented. Since potassium, magnesium and sulfate were already ruled out as the limiting nutrient, and ammonium nitrogen (1 g/L) is abundant in the updated CSL medium, the focus is shifted towards micronutrients. Individual constituents of the Syn-6-medium were supplemented to the established CSL medium, which contained 1 g/L (NH_4_)_2_SO_4_ and was prepared with the CSL stored for 23 months. The results are presented in Fig. 7.

In Fig. 7A, the impact of microelements, vitamins, trace elements and amino acids solutions, originated from Syn-6-MES medium, on the OTR of *O. polymorpha* is presented. For reference, cultivation on the CSL medium containing 16.5-months-old CSL and 1 g/L (NH_4_)_2_SO_4_ was plotted in Fig. 7A.

The Syn-6-MES medium consists of a basic salt solution containing KH_2_PO_4_, MgSO_4_, KCl, NaCl and calcium chloride CaCl_2_. Each of these “salt” components was supplemented to the CSL medium, but no impact of their addition was observed (Add. File 13). This confirms the assumption that macronutrients did not become unavailable with longer CSL storage time.

Figure 7A shows that the reference CSL cultivation prepared with 16.5-months-old CSL led to a maximum OTR of 38 mmol/L/h. With a longer CSL storage time of 23 months, the OTR was reduced to 24 mmol/L/h. The addition of selected vitamins and trace elements did not significantly improve the OTR of *O. polymorpha*. Together with the non-supplemented CSL medium, the OTR measured for vitamins and trace elements supplementation showed a shoulder after 7.2 h. The shoulder may indicate the consumption of overflow metabolites, ethanol and acetate, produced during the first cultivation hours. This shoulder is not apparent in the reference cultivation using the CSL stored for 16.5 months. Supplementing amino acids accelerated the growth of *O. polymorpha* between hours 3–22, to the same rate as the reference CSL stored for 16.5 months. A maximum OTR of 28 mmol/L/h was reached. Finally, supplementing microelements led to a significantly elevated OTR of 34 mmol/L/h, which corresponds to 90% of the maximum OTR reached by the reference CSL cultivation using CSL stored for 16.5 months.

To identify which metal constituents of the microelements solution were responsible for the improvement, each metal constituent was separately supplemented to the CSL medium. As depicted in Fig. 7A, all metals were qualitatively tested, as there is a high probability of them being precipitated as scarcely soluble sulfides. These metal ions serve as cofactors for specific enzymes. Thus, after the relevance of these metal ions had been proved, these metal ions were investigated in more detail. An increase of OTR, as well as biomass production, indicates an improvement brought about by the metal ions. The results of this targeted supplementation are shown in Fig. 7B. The addition of MnSO_4_ did not significantly improve the OTR of *O. polymorpha*. On the other hand, supplementing FeSO_4_ and ZnSO_4_ increased the OTR by up to 13%, compared to the non-supplemented CSL medium. Adding CuSO_4_ showed the highest effect, by increasing the OTR to 30 mmol/L/h, which corresponded to 80% of the max. OTR reached by the reference CSL cultivation using CSL stored for 16.5 months.

Figure 7C compares the resulting offline analytical data TO and CDW for different CSL media. The total oxygen consumed by the reference CSL medium prepared with 16.5-months-old CSL, 23-months-old CSL, as well as the 23-months-old CSL supplemented with microelements was similar, ranging between 95 and 103 mmol/L. With CSL stored for 16.5 months, a high final CDW of 3.8 g/L was obtained. With longer CSL storage of 23 months, CDW was drastically reduced by 28% to 2.7 g/L. The addition of microelements to the CSL medium prepared with 23-months-old CSL improved the CDW to 3.6 g/L. This corresponds to 95% of the previous CDW value obtained with the reference CSL.

Thus, out of all constituents of the microelements solution, CuSO_4_ showed to be the most limiting nutrient in the CSL batch stored for 23 months. This may be explained by the fact that copper ions play an important role as cofactor in metalloenzymes, as well as in electron transport and defense mechanisms against oxidative stress [79–81]. With their key role in cellular respiration and activity of cytochrome C oxidase, the supplementation of copper ions may, thus, be critical for *O. polymorpha*. This can be seen by the increased OTR brought about by CuSO_4_ supplementation. Furthermore, a loss of FeSO_4_ and ZnSO_4_ was observed, while MnSO_4_ appears to be less affected with longer storage times. However, even though supplementing these individual constituents resulted in an OTR improvement, the greatest impact was achieved by supplementing a mixture of these microelements. It is widely reported that microelements such as trace metals act as essential substrates or cofactors for many enzymes, and, thus, are critical for cellular metabolism [82–85]. Others have reported that iron, for example, may be a limiting nutrient which is detrimental for cellular growth in *Bacillus subtilis* and *Escherichia coli* [86, 87]. However, an excess metals exposure would result in microbial toxicity [88]. Jones and Gadd [88] reported that complex fermentation substrates, such as molasses and corn steep liquor, have the ability to tightly bind metal ions and can, therefore, affect their overall availability in the medium. In addition, yeasts are reported to form hydrogen sulfide (H_2_S), which binds metals and precipitates them into insoluble sulfides, such as copper sulfide [1]. Therefore, the precipitation of essential microelements is assumed to be magnified with longer CSL storage times. Based on the presented supplementation results, it should be considered to routinely complement culture media with complex ingredients with a standardized amount of metal ions.

CSL media in this work were prepared using samples of crude CSL stocks that were heat sterilized and stored at 4 °C. The original crude CSL stocks were also stored at 4 °C. However, the crude CSL stocks were not collected from the designated sources under 100% sterile conditions, thus, microbial activity should also be expected even during cool storage. Since the sealed CSL container represents anaerobic conditions, very slow metabolic activity of the contaminating microorganisms may occur, metabolizing sulfate in the CSL and forming H_2_S. H_2_S, in turn, produces insoluble sulfides with the trace elements in the CSL, which highly likely forms a sediment. Thus, if samples are taken from the CSL stock and the container is not sufficiently shaken beforehand, there will be little to no trace elements in that sample. The longer the CSL stock is stored, the more pronounced this effect probably is.

## Conclusions

In this study, a fast and reliable CSL screening method was developed by using the RAMOS system designed for shake flasks and 96-well microtiter plates. The parallel measurements by the RAMOS system showed an excellent reproducibility, given that the standard deviations are hardly if at all visible. For *O. polymorpha* cultivations, CSL proved to be a suitable alternative nutrient source that can replace costly yeast extract and peptone. For the employed CSL media, glucose was chosen as a carbon source to focus on the suitability and versatility of CSL as an alternative complex nutrient source. Literature reported that wildtype strains of *O. polymorpha* can utilize less accessible sugars, such as cellobiose and xylose [89]. Therefore, it can be assumed with some confidence that the CSL-based medium can also be employed in combination with these sugars. The combination of CSL and these other less accessible carbon sources may be investigated in further studies. Based on OTR and OD_600_ measurements, *O. polymorpha* exhibited less sensitivity to batch-to-batch variation in CSL, compared to *E. coli*, as shown in our previous publication. For the employed CSL-glucose medium, containing 2.5 g d.s/L CSL and 5 g/L glucose, a supplementation of at least 0.5 g/L (NH_4_)_2_SO_4_ is required, to avoid nitrogen limitation in *O. polymorpha* cultivations. Nitrogen limitation can be bypassed by increasing the ratio of CSL to glucose in the medium. In addition, nitrogen limitation resulted in a morphological change of *O. polymorpha*, rendering OD_600_ measurements unreliable. Future studies should incorporate metabolomic analysis, to further validate the underlying phenomena and results. The RAMOS system could detect deteriorating CSL performance associated with extended CSL storage times, caused by possible precipitation of micronutrients. Incomplete or lack of sterilization of crude CSL stocks could also lead to slow microbial activity, forming insoluble compounds with trace elements present in the CSL. In future studies, more investigations should be performed towards identifying the specific microbiomes in the different CSL variants, as these are likely to slowly degrade specific nutrients in the CSL. Through targeted supplementation, the missing micronutrients (copper, iron and zinc) could be identified and replenished. This micronutrient supplementation is assumed to likely be cost-competitive, if the micronutrients to be supplemented can be limited to a few, against composing a fully defined fermentation media containing a plethora of pure compounds. Given the nature of CSL as an agricultural side stream, this supplementation approach also gives an indirect value for the sustainability and circularity by using CSL. Additionally, analysis of key enzymes, such as alcohol oxidase activity, may be integrated in further studies, to gain more understanding of the metabolic state of *O. polymorpha*. To predict the success of future large-scale fermentation processes and eliminate poorly performing CSL batches, the application of the RAMOS system for CSL quality control is recommended. Now that it is clear that complex media components, such as CSL, can degrade over time, in the future, the limiting components should be systematically analyzed in relation to the microbes used, as well as the storage technique and duration of storage.

## Electronic supplementary material

Below is the link to the electronic supplementary material.

Supplementary Material 1

## Data Availability

The datasets supporting the conclusions of this article are included within the article and its supplementary information files.

## Abbreviations

*B. subtilis*: *Bacillus subtilis*
CSL: Corn steep liquor
*E. coli*: *Escherichia coli*
GFP: Green fluorescent protein
HPLC: High pressure liquid chromatography
MTP: Microtiter plate
OD_600_: Optical density at 600 nm
maximum OTR: Highest value of measured oxygen transfer rate (mmol/L/h)
OTR: Oxygen transfer rate (mmol/L/h)
OTR_max_: Maximum oxygen transfer capacity (mmol/L/h)
RAMOS: Respiration Activity Monitoring System device for shake flasks
*O. polymorpha*: *Ogataea polymorpha*
TO: Total oxygen (mmol/L)
UPLC: Ultra-performance liquid chromatography
YPD medium: Yeast extract-peptone-dextrose medium
µTOM: Micro(µ)-scale Transfer rate Online Measurement device for 96-deepwell microtiter plates
