# Supplementary material for: Metabolic studies of Ogataea polymorpha using nine different corn steep liquors
Source: BMC Biotechnol. 2025 Jan 10;25:5. doi: 10.1186/s12896-024-00927-5 (PMC11724537; doi:10.1186/s12896-024-00927-5)
Supplement: Supplementary file 1 — Supplementary Material 1 [file 12896_2024_927_MOESM1_ESM.docx]

**Metabolic studies of *Ogataea polymorpha* using nine different corn steep liquors**

Sekar Mayang W Wahjudi^a^, Dominik Engel^a^, and Jochen Büchs^a,*^

^a^ Aachener Verfahrenstechnik – Biochemical Engineering, RWTH Aachen University, Forckenbeckstr. 51, 52074 Aachen, Germany

^*^ Corresponding author:

Prof. Dr.-Ing. Jochen Büchs, RWTH Aachen University, Aachener Verfahrenstechnik – Biochemical Engineering, Bldg. NGP2/A-307, Forckenbeckstr. 51, 52074 Aachen, Germany; Phone: +49 (0) 241 – 80-24633; Fax: +49 (0) 241 80 22635; E-mail: jochen.buechs@avt.rwth-aachen.de

**Additional Material**





**Additional File 1: Comparison of lactic acid and acetic acid content in varying CSL variants.**





**Additional File 2: Free primary amino acids content in varying CSL variants.**





**Additional File 3: Total nitrogen content in varying CSL variants.**





**Additional File 4: Total phosphorus in varying CSL variants.**

| **CSL variant** | **Composition (% w/w on dry basis)** | | | |
| --- | --- | --- | --- | --- |
|  | **Lactic acid** | **Acetic acid** | **Total nitrogen** | **Total phosphorus** |
| **1** | 33.2 | 0.0 | 6.7 | 3.5 |
| **2** | 29.0 | 0.1 | 7.1 | 3.7 |
| **3** | 29.0 | 0.2 | 6.9 | 3.4 |
| **4** | 24.0 | 0.0 | 6.3 | 3.3 |
| **5** | 21.0 | 0.0 | 6.5 | 3.0 |
| **6** | 35.4 | 0.0 | 6.7 | 3.3 |
| **7** | 29.8 | 1.2 | 7.5 | 3.4 |
| **8** | 34.0 | 1.2 | 7.8 | 3.2 |
| **9** | 33.5 | 1.4 | 7.9 | 3.2 |

**Additional File 5: Concentration of lactic acid, acetic acid, total nitrogen and total phosphorus in the nine different CSL variants.**





**Additional File 6: Growth of *Ogataea polymorpha* on supplemented CSL medium, using CSL batch 1 and magnesium sulfate heptahydrate (calculated as magnesium sulfate) in a µTOM device.**(A) Oxygen transfer rate of *O. polymorpha* RB11 pC10-FMD (P_FMD_-GFP). OTR curves of different magnesium sulfate heptahydrate (calculated as magnesium sulfate) supplementations. Data points shown are mean values measured from 4 - 6 individual wells. Shadows, indicating the respective standard deviation, are barely visible, caused by the low standard deviations < 0.5. (B) Optical density at 600 nm wavelength (OD_600_) and total oxygen (TO), measured after 20 h. OD_600_ was each obtained from 2 individual wells. TO values were calculated from OTRs in 5 - 6 replicates. Error bars in (B) for TO values indicate the respective standard deviation. Experimental parameters: round deep-well 96-well microtiter plate (MTP), CSL 1 concentration $c_{CSL,1}$ = 2.5 g dry substance/L, glucose concentration $c_{glucose}$= 5 g/L, ammonium sulfate concentration $c_{MgSO_{4}}$= 0 – 0.5 g/L, MES buffer concentration $c_{MES}$ = 0.1 M, initial pH = 6.0, culture volume V_L_ = 200  μL/well, shaking frequency n = 350 rpm, shaking diameter d_0_ = 50 mm, humidity = 80 %, temperature T = 37 °C and initial OD_600_ = 0.1.





**Additional File 7: Growth of Ogataea polymorpha on supplemented CSL medium, using CSL batch 1 and potassium dihydrogen phosphate in RAMOS flasks.** (A) Oxygen transfer rate of O. polymorpha RB11 pC10-FMD (P_FMD_-GFP). OTR curves of different potassium dihydrogen phosphate supplementations. Data points shown are mean values, ± half the amplitude between duplicate cultivations is shown in shadows. (B) Optical density at 600 nm wavelength (OD_600_) and total oxygen (TO), measured after 20 h. OD_600_ was obtained in triplicates, whereas the mean value for TO was calculated from two OTR values from duplicate cultivation. Error bars for OD_600_ values in (B) indicate the respective standard deviation. Experimental parameters: 250 mL RAMOS flask, CSL 1 concentration $c_{CSL,1}$ = 2.5 g dry substance/L, glucose concentration $c_{glucose}$= 5 g/L, potassium dihydrogen phosphate concentration $c_{KH_{2}PO_{4}}$= 0 – 1.0 g/L, MES buffer concentration $c_{MES}$ = 0.1 M, initial pH = 6.0, culture volume V_L_ = 10 mL, shaking frequency n = 350 rpm, shaking diameter d_0_ = 50 mm, temperature T = 37 °C and initial OD_600_ = 0.1.





**Additional File 8: Growth of *Ogataea polymorpha* on supplemented CSL medium, using CSL batch 4 and ammonium sulfate in a µTOM device.** (A) Oxygen transfer rate of *O. polymorpha* RB11 pC10-FMD (P_FMD_-GFP). OTR curves of different ammonium sulfate supplementations. Data points shown are mean values measured from 4 - 6 individual wells. Shadows, indicating the respective standard deviation, are barely visible, caused by the low standard deviations < 0.5. (B) Optical density at 600 nm wavelength (OD_600_) and total oxygen (TO), measured after 20 h. OD_600_ was each obtained from 2 individual wells. TO values were calculated from OTRs in 5 - 6 replicates. Error bars in (B) for TO values indicate the respective standard deviation. Experimental parameters: round deep-well 96-well microtiter plate (MTP), CSL 4 concentration $c_{CSL,4}$ = 2.5 g dry substance/L, glucose concentration $c_{glucose}$= 5 g/L, ammonium sulfate concentration $c_{(NH_{4})_{2}SO_{4}}$= 0 – 0.7 g/L, MES buffer concentration $c_{MES}$ = 0.1 M, initial pH = 6.0, culture volume V_L_ = 200  μL/well, shaking frequency n = 350 rpm, shaking diameter d_0_ = 50 mm, humidity = 80 %, temperature T = 37 °C and initial OD_600_ = 0.1.





**Additional File 9: Cell size distribution of *Ogataea polymorpha* cultivations in a µTOM device at t_0_ (0 h).** Cell count distribution at different *O. polymorpha* cell diameters obtained after sampling at the beginning of the cultivation. Experimental parameters: 250 mL RAMOS flask, CSL 1 concentration $c_{CSL,1}$= 2.5 g dry substance/L, glucose concentration $c_{glucose}$ = 5 g/L, ammonium sulfate concentration $c_{(NH_{4})_{2}SO_{4}}$ = 0 and 0.6 g/L, MES buffer concentration $c_{MES}$ = 0.1 M, initial pH = 6.0, culture volume V_L_ = 10 mL, shaking frequency n = 350 rpm, shaking diameter d_0_ = 50 mm, temperature T = 37 °C and initial OD_600_ = 0.1


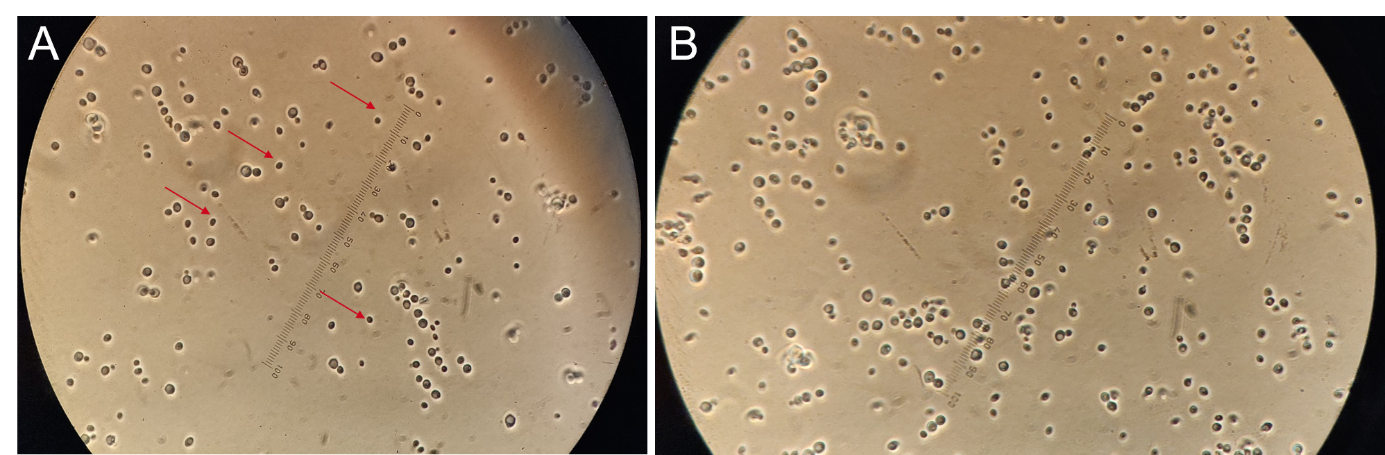


**Additional File 10: Microscopic picture of *Ogataea polymorpha* at the end of cultivation (8.5 h) in Fig. 5.** (A) Picture taken from the *O. polymorpha* RB11 pC10-FMD (P_FMD_-GFP) cultivation with 0 g/L ammonium sulfate ((NH_4_)_2_SO_4_). (B) Picture taken from the *O. polymorpha* RB11 pC10-FMD (P_FMD_-GFP) cultivation with 0.4 g/L ammonium sulfate ((NH_4_)_2_SO_4_). Red arrows in (A) indicate microcells produced under ammonium limited condition.





**Additional File 11: Influence of ammonium limitation on the biomass production of Ogataea polymorpha in RAMOS flasks**. (A) Oxygen transfer rate of O. polymorpha RB11 pC10-FMD (PFMD-GFP). Data points shown were each measured once. (B) Optical density at 600 nm wavelength (OD_600_), total oxygen (TO) and final pH values, measured after 14 h. OD_600_ and CDW values were obtained in triplicates. Error bars for OD_600_ in (B) indicate the respective standard deviation. Experimental parameters: 250 mL RAMOS flask, CSL 1 concentration $c_{CSL,1}$ = 2.5 g dry substance/L, glucose concentration $c_{glucose}$ = 5 g/L, ammonium sulfate concentration $c_{(NH_{4})_{2}SO_{4}}$ = 0 and 0.4 g/L, MES buffer concentration $c_{MES}$ = 0.1 M, initial pH = 6.0, culture volume V_L_ = 10 mL, shaking frequency n = 350 rpm, shaking diameter d_0_ = 50 mm, temperature T = 37 °C and initial OD_600_ = 0.1.





**Additional File 12: Decreasing CSL performance with elevated storage times in *Ogataea polymorpha* cultivation in a µTOM device.** Oxygen transfer rate of *O. polymorpha* RB11 pC10-FMD (P_FMD_-GFP) on CSL media containing CSL stored for different durations. Data points shown are mean values measured from 4 - 6 individual wells. Shadows, indicating the respective standard deviation, are barely visible, caused by the low standard deviations < 0.5. CSL stocks were stored at 4 °C. Experimental parameters: round deep-well 96-well microtiter plate (MTP), CSL 1 concentration $c_{CSL,1}$= 2.5 g dry substance/L, glucose concentration $c_{glucose}$ = 5 g/L, MES buffer concentration $c_{MES}$ = 0.1 M, initial pH = 6.0, culture volume V_L_ = 200 μL/well, shaking frequency n = 350 rpm, shaking diameter d_0_ = 50 mm, humidity = 80 %, temperature T = 37 °C and initial OD_600_ = 0.1.





**Additional File 13: Influence of salts on the decreased CSL performance at 23 months shelf life in a µTOM device.** Oxygen transfer rate of *Ogataea polymorpha* RB11 pC10-FMD (P_FMD_-GFP) on CSL medium enriched with different supplements. The reference CSL medium as well as all the other media contained 1.0 g/L ammonium sulfate. The storage time of the reference CSL was 16.5 months at 4 °C, whereas the CSL employed in all other media was stored for 23 months at 4 °C. Data points shown are mean values measured from 4 - 6 individual wells. Shadows, indicating the respective standard deviation, are barely visible, caused by the low standard deviations < 0.5. Experimental parameters: round deep-well 96-well microtiter plate (MTP), CSL 1 concentration $c_{CSL,1}$ = 2.5 g dry substance/L, glucose concentration $c_{glucose}$= 5 g/L, ammonium sulfate concentration $c_{(NH_{4})_{2}SO_{4}}$ = 1.0 g/L, MES buffer concentration $c_{MES}$ = 0.1 M, initial pH = 6.0, culture volume V_L_ = 200 μL/well, shaking frequency n = 350 rpm, shaking diameter d_0_ = 50 mm, humidity = 80 %, temperature T = 37 °C and initial OD_600_ = 0.1.
